# Rictor positively regulates B cell receptor signaling by modulating actin reorganization via ezrin

**DOI:** 10.1371/journal.pbio.2001750

**Published:** 2017-08-18

**Authors:** Lu Huang, Yongjie Zhang, Chenguang Xu, Xiaomei Gu, Linlin Niu, Jinzhi Wang, Xiaoyu Sun, Xiaoming Bai, Xingtian Xuan, Qubei Li, Chunwei Shi, Bing Yu, Heather Miller, Gangyi Yang, Lisa S. Westerberg, Wanli Liu, Wenxia Song, Xiaodong Zhao, Chaohong Liu

**Affiliations:** 1 Chongqing Key Laboratory of Child Infection and Immunity, Children’s Hospital of Chongqing Medical University, Chongqing, China; 2 Department of Pediatric Research Institute, Children’s Hospital of Chongqing Medical University, Chongqing, China; 3 Ministry of Education Key Laboratory of Child Development and Disorders, Children’s Hospital of Chongqing Medical University, Chongqing, China; 4 MOE Key Laboratory of Protein Sciences, Collaborative Innovation Center for Diagnosis and Treatment of Infectious Diseases, School of Life Sciences, Institute for Immunology, Tsinghua University, Beijing, China; 5 Children’s Hospital Respiratory Center of Chongqing Medical University, Chongqing, China; 6 Department of Pathogen Biology, School of Basic Medicine, Huazhong University of Science and Technology, Wuhan, China; 7 Department of Intracellular Pathogens, National Institute of Allergy and Infectious Diseases, Hamilton, Montana, United States of America; 8 Department of Endocrinology, the Second Affiliated Hospital, Chongqing Medical University, Chongqing, China; 9 Department of Microbiology Tumor and Cell Biology, Karolinska Institutet, Stockholm, Sweden; 10 Department of Cell Biology and Molecular Genetics, University of Maryland, College Park, Maryland, United States of America; The Scripps Research Institute, United States of America

## Abstract

As the central hub of the metabolism machinery, the mammalian target of rapamycin complex 2 (mTORC2) has been well studied in lymphocytes. As an obligatory component of mTORC2, the role of Rictor in T cells is well established. However, the role of Rictor in B cells still remains elusive. Rictor is involved in B cell development, especially the peripheral development. However, the role of Rictor on B cell receptor (BCR) signaling as well as the underlying cellular and molecular mechanism is still unknown. This study used B cell–specfic Rictor knockout (KO) mice to investigate how Rictor regulates BCR signaling. We found that the key positive and negative BCR signaling molecules, phosphorylated Brutons tyrosine kinase (pBtk) and phosphorylated SH2-containing inositol phosphatase (pSHIP), are reduced and enhanced, respectively, in Rictor KO B cells. This suggests that Rictor positively regulates the early events of BCR signaling. We found that the cellular filamentous actin (F-actin) is drastically increased in Rictor KO B cells after BCR stimulation through dysregulating the dephosphorylation of ezrin. The high actin-ezrin intensity area restricts the lateral movement of BCRs upon stimulation, consequently reducing BCR clustering and BCR signaling. The reduction in the initiation of BCR signaling caused by actin alteration is associated with a decreased humoral immune response in Rictor KO mice. The inhibition of actin polymerization with latrunculin in Rictor KO B cells rescues the defects of BCR signaling and B cell differentiation. Overall, our study provides a new pathway linking cell metablism to BCR activation, in which Rictor regulates BCR signaling via actin reorganization.

## Author summary

As the central hub of cell metabolism, the mammalian target of rapamycin complex (mTORC) integrates immune signals and metabolic cues for the maintenance and activation of these systems. Rictor is the core component of the mammalian target of rapamycin complex 2 (mTORC2), and loss of this protein leads to an immunodeficiency that involves (among other things) impaired antibody production. B cell receptor (BCR) signaling is critical for antibody generation and although it has been shown that loss of Rictor in B cells negatively impacts this function, the underlying molecular mechanisms are unknown. Here, we show that both early and distal BCR signaling is reduced in Rictor knockout (KO) B cells. We find that the reduction in BCR signaling stems from defective clustering of BCRs during early B cell activation. This seems to be caused by the uncontrolled activation of the actin-connecting protein ezrin, which leads to a rigid actin fence that restricts the lateral movement of BCRs in the membrane. Interestingly, treatment of Rictor KO mice with an actin inhibitor rescues the BCR signaling. Our findings suggest that Rictor helps to allow effective BCR signaling in B cells by triggering reorganization of the actin network, thereby enabling an appropriate antibody response during infection.

## Introduction

B cell receptor (BCR) signaling is vital for B cell development and function. When BCRs are cross-linked by antigens, it induces the conformational changes of signaling subunits immunoglobulin α chain (Igα) and immunoglobulin β chain (Igβ). The conformational changes of Igα and Igβ lead to the phosphorylation of immunoreceptor tyrosine-based activation motif (ITAM) domains of Igα and Igβ. The phosphorylated ITAM domain recruits LYN proto-oncogene, Src family tyrosine kinase (Lyn) for phosphorylation, and the phosphorylation of Lyn activates spleen tyrosine kinase (Syk). This initiates the activation of downstream signaling cascades, such as the activation of Brutons tyrosine kinase (Btk) and phospholipase C gamma 2 (PLCγ2) [1–3]. At the end of activation of BCR signaling, the negative regulators of BCR signaling are also activated, such as phosphorylated SH2-containing inositol phosphatase (pSHIP), which is regulated by Lyn [4–7]. Recently, with the development of the high-resolution technique of total internal reflection fluorescent microscopy (TIRFm), the molecular details of the initiation events in BCR activation have been revealed [8–10]. The conformational changes of the BCR expose the Cμ4 domain of membrane immunoglobulin M (IgM) for BCR self-aggregation [11] and ITAMs for signaling molecules to bind [12].

The role of actin on BCR signaling has been characterized recently with TIRFm as well. Actin is known to be involved in BCR capping [13,14], and the disruption of actin delays the attenuation of BCR signaling in B cells induced by soluble antigen (sAg) [15] or even induces BCR signaling alone, without antigen stimulation [16]. TIRFm coupled with single-molecule tracking techniques has dissected the underlying mechanism that links the actin network and BCR movement. In resting B cells, actin and ezrin together form a network that both defines compartments containing mobile BCRs and establishes boundaries restricting BCR diffusion. The BCR diffusion coefficient is inversely related to the actin intensity on the plasma membrane. Breaking down of the actin fence by latruculin treatment increases the diffusion coefficient of BCRs and induces BCR signaling comparable to that triggered by BCR cross-linking [17,18]. Therefore, actin depolymerization is essential for the initiation of BCR signaling.

As a core component of mTORC2, Rictor has been studied recently in all kinds of cells. Although Rictor deletion early in B cells using vav guanine nucleotide exchange factor (*Vav*)-Cre has a modest effect on the development of pro- and pre-B cells in the bone marrow by up-regulating forkhead box O1 (FoxO1) and recombination activating 1 (Rag-1) [19,20], it causes a severe impact on the peripheral development [19]. The reduction of marginal zone (MZ) B cells and B1a cells is more severe than folicular (FO) B cells. Furthermore, antibody production is greatly impaired when mature B cells lose Rictor expression after completing their development by using Cre-ER^T2^*Rictor*^fl/fl^ mice[19]. Mechanistically, Rictor is vital for the induction of prosurvival genes, suppression of proapoptotic genes, nuclear factor κB (NF-κB) induction after BCR activation, and nuclear factor κB2/p52 generation [19]. Therefore, Rictor is critical for B cell survival signals initiated via Phosphotidylinositol 3 kinase (PI3K) [19]. However, it is unknown how Rictor affects BCR signaling or early B cell activation.

The activation of both BCR and T cell receptor (TCR) induces the dephosphorylation of ezrin-radixin-moesin (ERM) proteins that are the linkers between the plasma membrane and the actin cytoskeleton and induces the detachment of ERM from the actin cytoskeleton [21–23]. Similar to the role of the actin cytoskeleton in the steady state, ezrin also forms a network that, together with actin, restricts the movement of BCRs and slows the diffusion rate [17]. The transient inactivation of ERM, such as dephosphorylation of ezrin, can increase the diffusion rate of unengaged BCRs. The dephosphorylation of ezrin can alter the interaction between the actin cystoskeleton and plasma membrane, which can in turn alter the B cell’s morphology by modulating the filopodia. Consequently, this impairs BCR clustering and B cell spreading during B cell activation [24]. The BCR-mediated phosphorylation of ezrin negatively regulates activation events such as the phosphorylation of tyrosine kinases [25]. In systemic lupus erythematosus (SLE) T cells, the binding of autoantibodies to the cluster of differentiation 3 (CD3)-TCR complex induces the phosphorylation of ezrin and actin polymerization [26]. The inhibition of ezrin with pharmacological inhibitors or small interfering RNA (siRNA) reduces the formation of actin stress fibers [27]. The phosphorylation of ezrin is regulated by serine/threonine kinases including rho-associated coiled-coil-containing protein kinase (ROCK) and protein kinase C (PKC) [28,29]. Considering that Rictor is involved in the reorganization of actin, it is not clear whether Rictor links ezrin to regulate BCR signaling as well as the underlying mechanism.

In this study, we used cluster of differentiation 19 (*cd19*)-Cre to delete Rictor specifically in B cells and excluded the deletion outside of the B lineage by using *Vav*-Cre and mixed chimerism in non–B lineages, irradiation-induced load of apoptotic bodies when generating chimera mice. We found that Rictor positively regulates BCR signaling via up-regulating Btk and down-regulating SH2-containing inositol phosphatase (SHIP). Mechanistically, the reduction of BCR signaling is caused by the less mobile BCRs in the activation state, and Rictor deficiency disrupts the early actin depolymerization phase during BCR activation and enhances the actin polymerization and phosphorylation of ezrin. All of these account for the high intensity of ezrin-actin areas that restrict the diffusion of BCRs, which are essential for the triggering of BCR signaling. Furthermore, the reduction of FO B cells was more severe in immunized Rictor KO mice, but we did not observe changes for MZ B cells. Interestingly, the introduction of Latrunculin B, an actin inhibitor *in vitro* and *in vivo*, can rescue the defect of differentiation of FO B cells and BCR signaling.

## Results

### Rictor is involved in BCR activation

**Fig 1:**
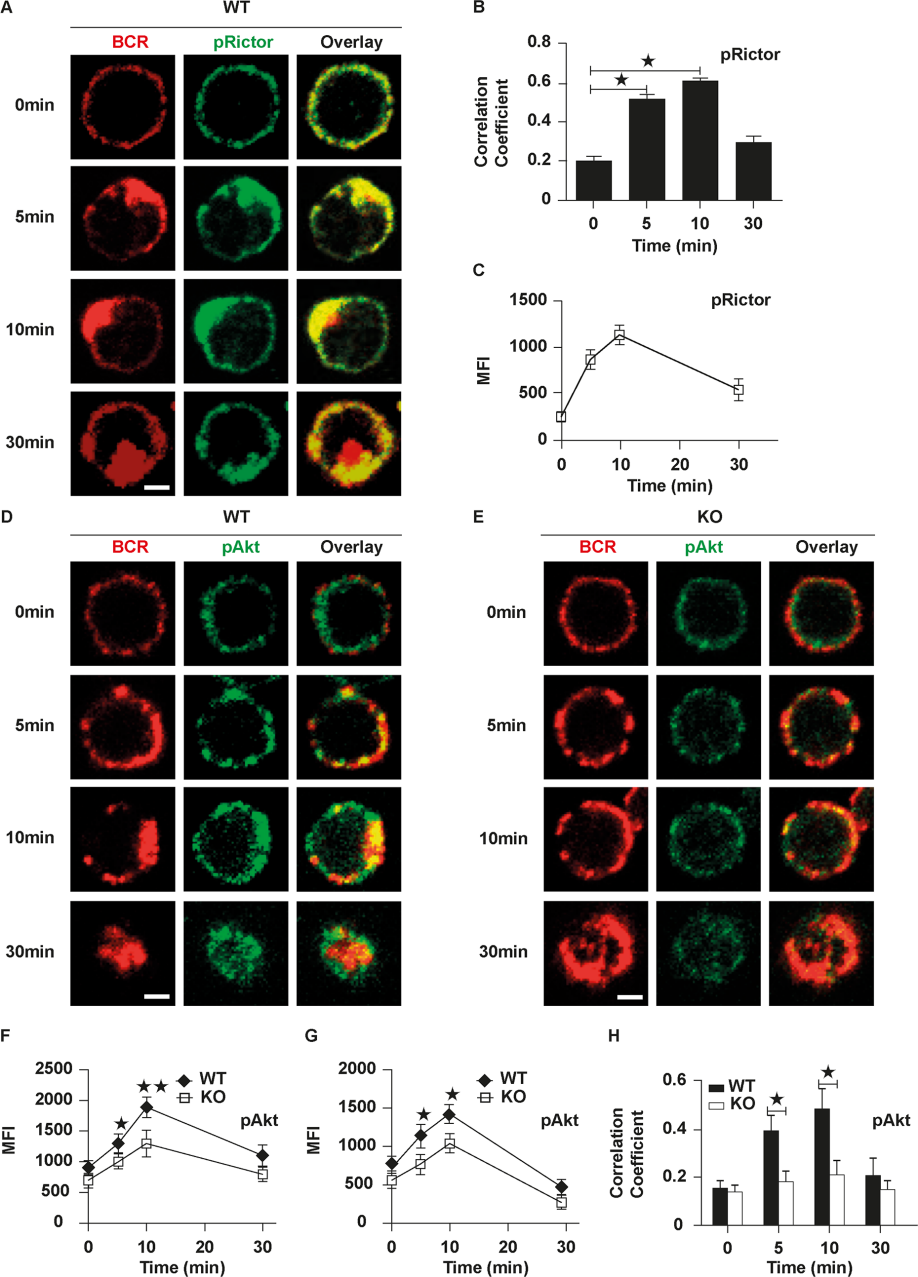
B cell receptor (BCR)-induced serine/threonine kinase (Akt) activation is inhibited in Rictor knockout (KO) B cells. To mimic soluble antigen (sAg), splenic wild-type (WT) or Rictor KO B cells were incubated with Alexa Fluor (AF) 546–monobiotinylated (mB)-Fab′–anti-immunoglobulin (Ig) for 10 min at 4°C to label the BCR. Then, the cells were either incubated with streptavidin or with the medium alone (0 min) as a control at 37°C for varying lengths of time. After fixation and permeabilization, the cells were stained for phosphorylated Rictor (pRictor) (A) or phosphorylated Akt (pAkt) (D and E) and analyzed using confocal microscopy (CFm). The mean fluorescence intensity (MFI) of pRictor (C) or pAkt (F) and the correlation coefficients (B and H) between the BCR and pRictor or pAkt were quantified using NIS-Elements AR 3.2 software. Flow cytometry analysis of MFI of pAkt after stimulation with sAg (G). Shown are representative images and mean values (±SD) from 3 independent experiments, in which over 50 cells were individually analyzed using NIS-Elements AR 3.2 software. Scale bars, 2.5 μm. Mann–Whitney *U* test was used to do the statistics, * *p* < 0.01. The numerical data (for B, C, E, F, G, and H) can be found in S1 Data.

To determine whether Rictor is involved in BCR activation or not, we examined the spatiotemporal relationship between BCR and Rictor using phosphorylated antibody specific for activated Rictor by confocal microscopy (CFm). At 0 min, phosphorylated Rictor (pRictor) was distributed on the plasma membrane evenly (Fig 1A). At 5 min and 10 min, pRictor was redistributed and cocapped with the BCR cluster (Fig 1A). At 30 min, the degree of cocapping of BCR with pRictor was decreased as BCRs started to be internalized (Fig 1A). We used a correlation coefficient to determine the colocalization of BCR and pRictor quantitatively. The colocalization between BCR and pRictor was increased over 10 min and decreased by 30 min. It increased significantly at 5 min and 10 min compared to 0 min (Fig 1B). Additionally, the levels of pRictor measured with mean fluroscence intensity (MFI) by NIS-Elements AR 3.2 software peaked at 10 min upon antigen stimulation (Fig 1C). These results suggest that Rictor is involved in BCR activation. In order to further determine whether Rictor signaling is also involved in BCR activation, we examined the location and expression levels of the downstream signaling molecule of Rictor, phosphorylated Akt (pAkt), in wild-type (WT) and Rictor KO B cells. First, in order to determine the deletion efficiency of *cd19-*Cre and line leakage, we examined the mRNA levels of *rictor* in B cells, CD4^+^, and CD8^+^ T cells using real time PCR (RT-PCR) and protein levels of Rictor in B cells using western blot. The mRNA levels of *rictor* and protein levels of Rictor were significantly lower in Rictor KO B cells but had no changes in CD4^+^ and CD8^+^ Rictor KO T cells (S1A and S1B Fig). This result suggests the *cd19-*Cre deletion efficiency is high in B cells without leakage in other types of immune cells. Similar to that of pRictor, the location of pAkt was distributed on the plasma membrane evenly at 0 min and cocapped with BCR at 5 min and 10 min and then began to have endocytosis at 30 min, together with BCR in WT B cells (Fig 1D). In contrast with that of WT B cells, the distribution of pAkt in Rictor KO B cells did not have obvious changes and BCR internalization was severely disrupted (Fig 1D and 1E). Additionally, the MFI of pAkt quantified with NIS-Elements AR 3.2 software in WT B cells was increased over 10 min and decreased by 30 min, and it was significantly decreased in KO B cells (Fig 1F). We also used flow cytomery to quantifiy the pAkt levels in WT and KO B cells after sAg stimulation and observed similar results as with CFm (Fig 1G). The colocalization of BCR with pAkt was increased by 10 min and decreased at 30 min but did not show obvious changes in WT B cells and was significantly decreased in KO B cells (Fig 1H). Taken together, these results suggest that Rictor as well as Rictor signaling is involved in BCR activation.

### The absence of Rictor down-regulates BCR signaling

**Fig 2:**
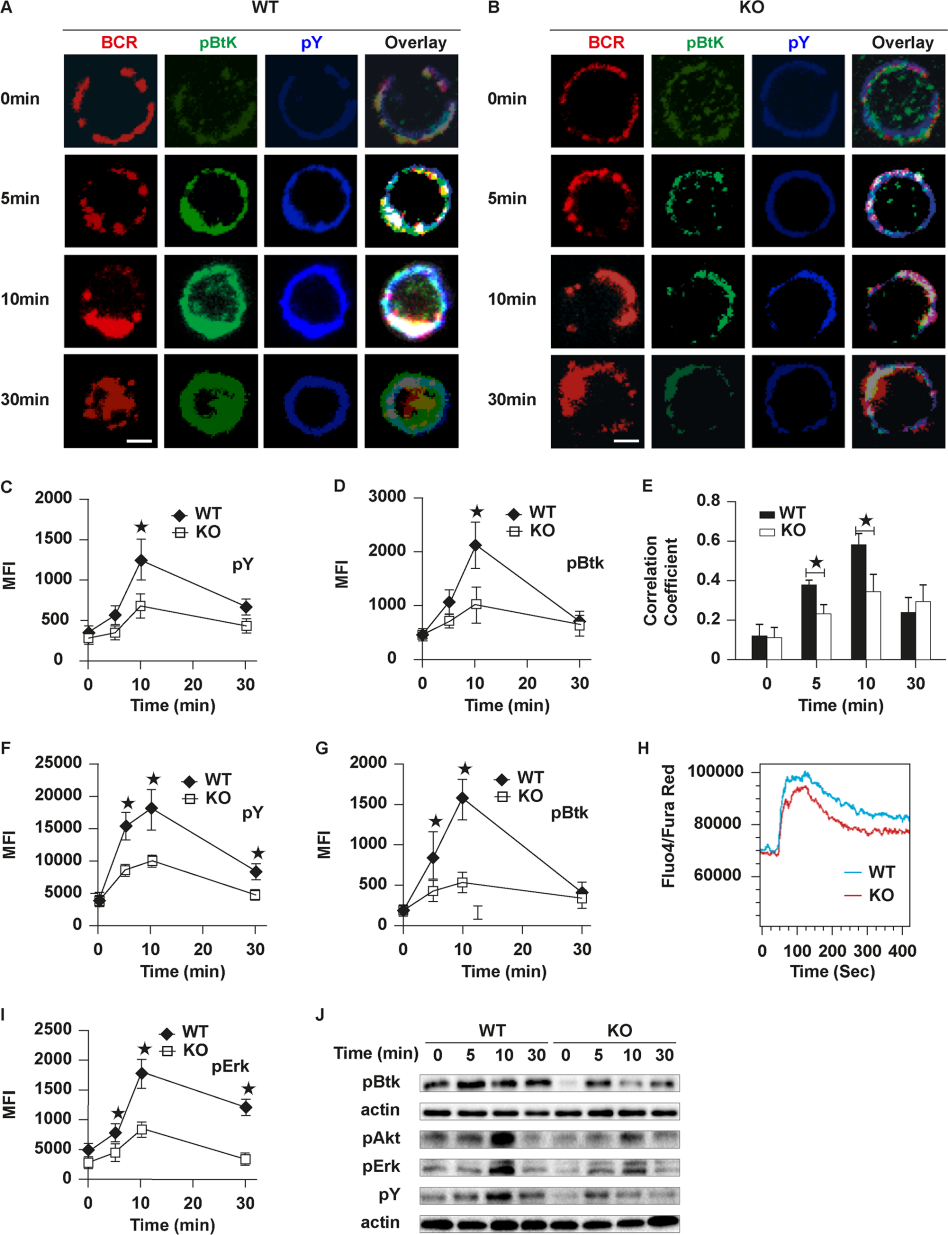
The levels of tyrosine and Brutons tyrosine kinase (Btk) phosphorylation in B cell receptor (BCR) clusters in response to soluble antigen (sAg) are reduced in Rictor knockout (KO) B cells. Splenic B cells were incubated with Alexa Fluor (AF) 546–monobiotinylated (mB)-Fab′–anti- immunoglobulin (Ig) without (−) or with streptavidin (sAg) at 4°C, washed, and warmed to 37°C for varying lengths of time. After fixation and permeabilization, the cells were stained for phosphotyrosine (pY) and phosphorylated Brutons tyrosine kinase (pBtk) and analyzed using confocal microscopy (CFm) or flow cytometry (A, B, F and G). The mean fluorescence intensity (MFI) of pY and pBtk were generated using NIS-Elements AR 3.2 software (C and D). The Pearson’s correlation coefficients between BCR and pY and pBtk staining in sAg-stimulated cells were determined using NIS-Elements AR 3.2 software (E). Flow cytometry analysis of the MFI of pY, pBtk, and phosphorylated extracellular regulated protein kinases (pErk) after stimulation with sAgs (F, G and I). Ca^2+^ flux analysis of splenic B cells activated with soluble monobiotinylated (mB)-Fab′–anti- immunoglobulin (Ig) plus streptavidin using flow cytometry (H). Western blot analysis of the pBtk, pAkt (Serine 473 [Ser473]), pErk, and pY (80 kDa) after stimulation with sAgs (J); actin was used as a loading control. Shown are representative images at indicated times and the average values (±SD) of about 50 cells from 3 independent experiments. Scale bars, 2.5 μm. Mann–Whitney *U* test was used to do the statistics, *p < 0.01. The numerical data (for C, D, E, F, G, and I) can be found in S1 Data.

**Fig 3:**
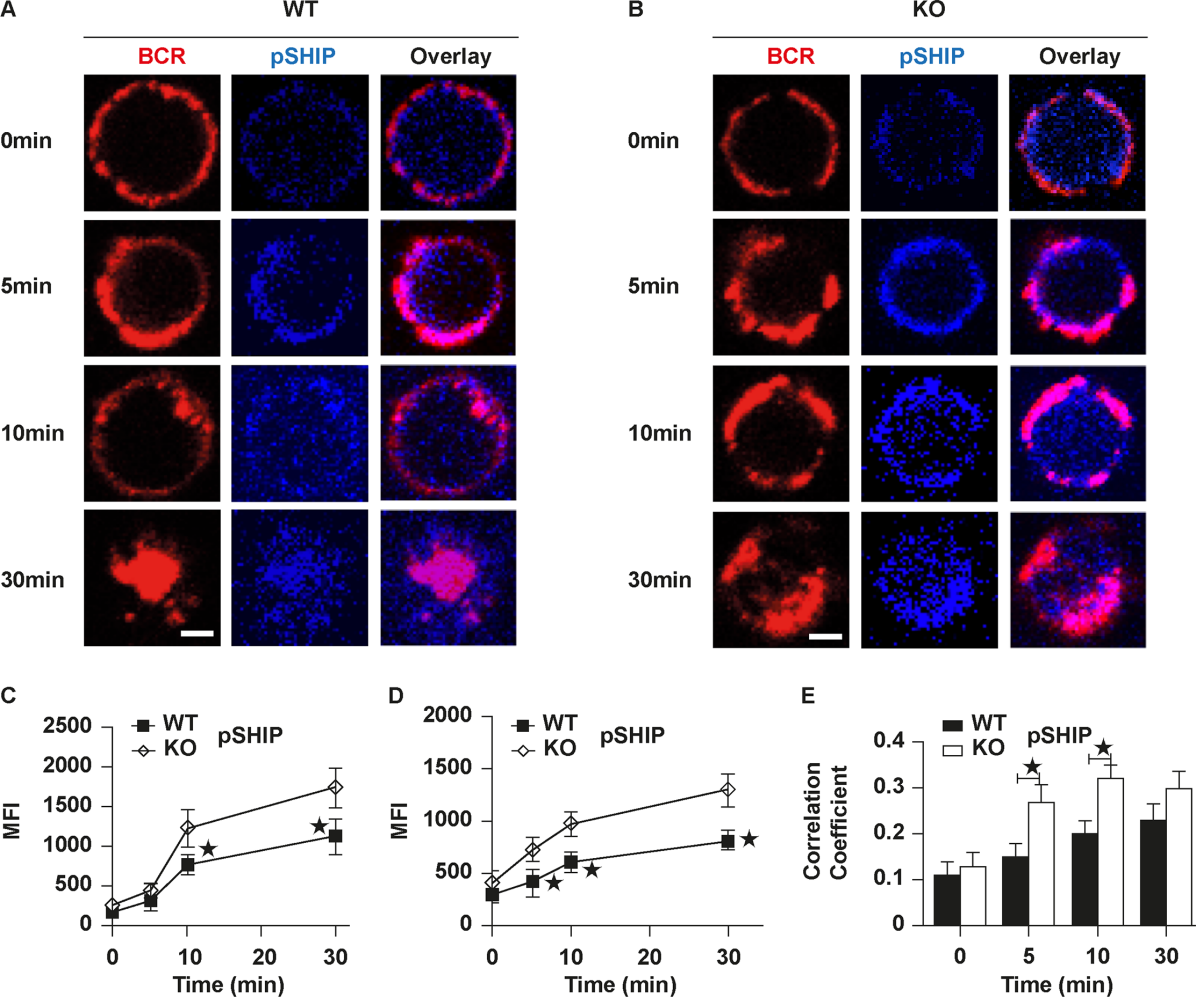
The recruitment of phosphorylated SH2-containing inositol phosphatase (pSHIP) to B cell receptor (BCR) clusters in B cells stimulated by soluble antigen (sAg) is increased in Rictor knockout (KO) B cells. Splenic B cells were incubated with Alexa Fluor (AF)546–monobiotinylated (mB)-Fab′–anti- immunoglobulin (Ig) without (−) or with streptavidin (sAg) at 4°C, washed, and warmed to 37°C for varying lengths of time. After fixation and permeabilization, the cells were stained for pSHIP and analyzed using confocal microscopy (CFm) or flow cytometry (A, B and D). The mean fluorescence intensity (MFI) of pSHIP was generated using NIS-Elements AR 3.2 software (C). Flow cytometry analysis of the MFI of pSHIP after stimulation with sAgs (D). The Pearson’s correlation coefficients between BCR and pSHIP staining in sAg-stimulated cells were determined using NIS-Elements AR 3.2 software (E). Shown are representative images at indicated times and the average values (±SD) of about 50 cells from 3 independent experiments. Scale bars, 2.5 μm. Mann–Whitney *U* test was used to do the statistics, *p < 0.01. The numerical data (for C, D, and E) can be found in S1 Data.

In order to determine the effect of Rictor deficiency on BCR signaling, we examined the levels of phosphorylated Brutons tyrosine kinase (pBtk) and pSHIP, the key postive and negative molecules of upstream BCR signaling, as well as total phosphotyrosine (pY) to indicate the total level of BCR signaling. The levels of pBtk and pY were increased over 10 min in WT B cells quantified by NIS-Elements AR 3.2 software and decreased by 30 min (Fig 2A, 2C and 2D). pBtk and pY were colocalized with BCR at 5 min and 10 min after stimulation and the degree of colocalization was decreased at 30 min in WT B cells (Fig 2A and 2E). In contrast to that of WT B cells, the levels of pBtk and pY were significantly decreased in KO B cells and the signalosomes of pBtk or pY were always distributed on the plasma membrane (Fig 2A–2D). The colocalization of pY and pBtk with BCR was increased over 10 min and decreased at 30 min in WT B cells, but it was dramatically decreased in KO B cells (Fig 2A, 2B and 2E). In order to further confirm the reduction of pY and pBtk in KO B cells, we examined the levels of pBtk and pY in WT and KO B cells stimulated by sAgs with flow cytometry. Similarly, we found the levels of pY and pBtk were significantly decreased in KO B cells (Fig 2F and 2G). Since the mammalian target of rapamycin (mTOR)/Akt and phospholipase C gamma 2 (PLCγ)/Ca^2+^ mobilization are seen as separate pathways downstream of the BCR, we examined the Ca^2+^ mobilization with flow cytometry. We found the Ca^2+^ mobilization was reduced in Rictor KO B cells after stimulation with sAg (Fig 2H). Additionally, we tested the distal BCR signaling levels such as phosphorylated extracellular regulated protein kinases (pErk) and we found that it was decreased in Rictor KO B cells (Fig 2I). To further confirm the down-regulation of BCR signaling by Rictor deficiency, we tested the levels of pBtk, pAkt, pErk, and pY with western blot after sAg stimulation and found a reduction in early and distal BCR signaling (Fig 2J). Furthermore, we examined the effect of Rictor deficiency on the recruitment of the negative signaling molecule, pSHIP. The MFI of pSHIP was increased over time until 30 min in WT B cells quantified by NIS-Elements AR 3.2 software, but it was significantly increased in KO B cells (Fig 3A–3C). Similar to the staining pattern of pY and pBkt (Fig 2A), pSHIP cocapped with BCR and went through internalization at 30 min in WT B cells (Fig 3A). In KO B cells, pSHIP was always colocalized with BCR (Fig 3B). To confirm the increase of pSHIP in KO B cells, we determined the levels of pSHIP in KO B cells by flow cytometry and found similar results (Fig 3D). We examined the colocalization of BCR and pSHIP using correlation coefficient in WT and KO B cells and found the colocalization of BCR and pSHIP was increased over time by 30 min in WT B cells and was significantly increased in Rictor KO B cells (Fig 3E). To exclude the effect of Rictor deficiency on BCR signaling that is due to B cell development, we examined the effect of Rictor deficiency on bone marrow and peripheral development in Rictor KO mice using flow cytometry. We found the frequency and number of pro-B cells were moderately increased and those of late pre-B cells and recirculating B cells were slightly decreased in Rictor KO mice (S2A–S2D Fig). We also examined the expression levels of interleukin 7 (IL-7) receptors and did not observe any changes (S2E Fig). Then, we examined the alteration of FO, MZ, and germinal center (GC) B cells in the spleen of Rictor KO mice without immunization. We found the frequency and number of FO and GC B cells was decreased and did not observe changes for MZ B cells (S3A–S3G Fig). Furthermore, we examined the expression levels of IgM and immunoglobulin D (IgD) and did not observe any differences of MFI of IgM and IgD between WT and KO B cells (S3H and S3I Fig). Overall, the deficiency of Rictor causes slight impact on the bone marrow and peripheral development. These results imply that Rictor regulates BCR signaling positively and the absence of Rictor leads to unbalanced positive and negative BCR signaling.

### The absence of Rictor leads to the enhanced polymerization of actin

**Fig 4:**
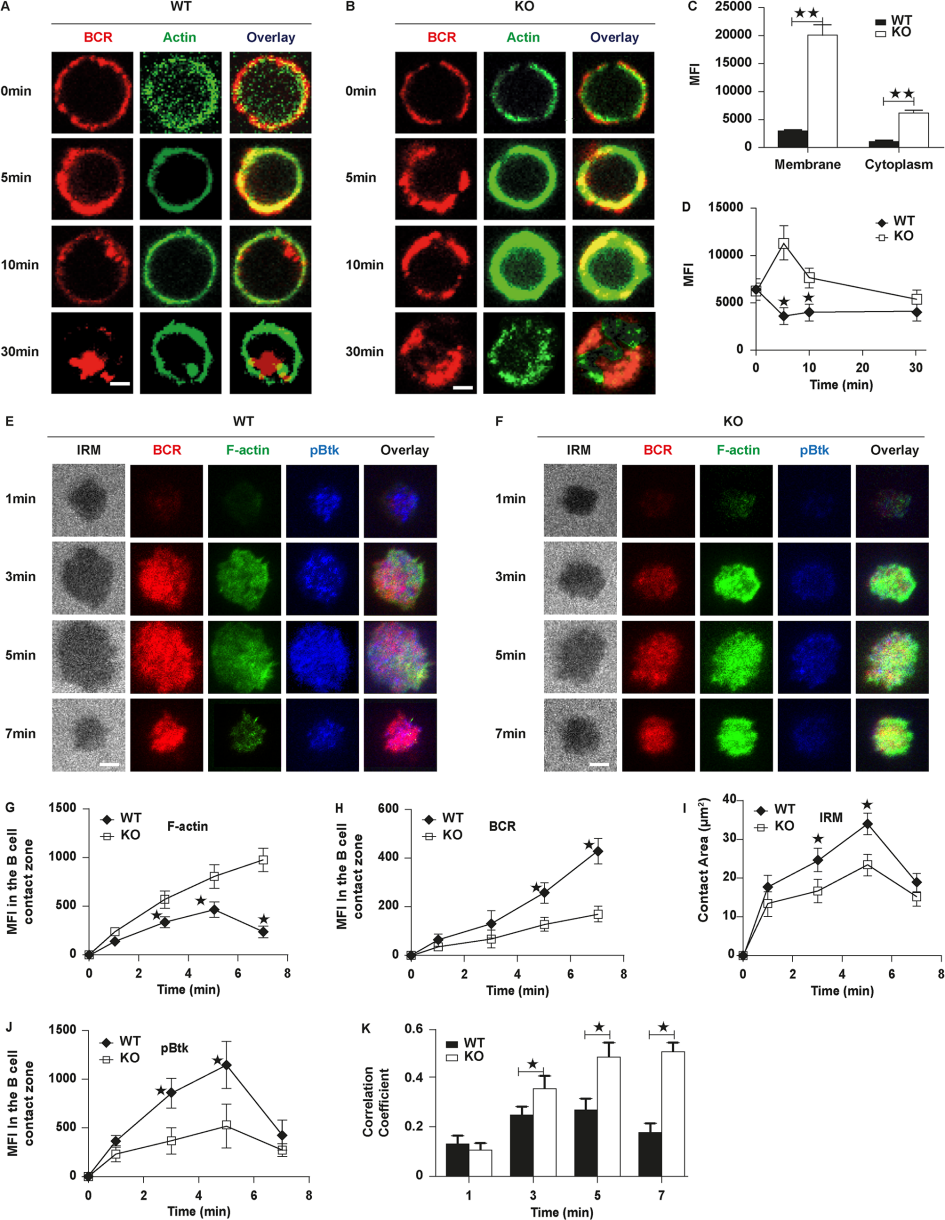
B cell receptor (BCR) cluster formation, B cell spreading, and BCR signalsome are reduced and actin polymerization is enhanced in Rictor knockout (KO) B cells. Splenic B cells were incubated with Alexa Fluor (AF) 546– monobiotinylated (mB)-Fab′–anti- immunoglobulin (Ig) without (−) or with streptavidin (sAg) at 4°C, washed, and warmed to 37°C for varying lengths of time. After fixation and permeabilization, cells were stained for Alexa Fluor 488 (AF488)-phallodin. Cells were analyzed using confocal microscopy (CFm) and flow cytometry. Shown are representative images at indicated times (A and B) and the average values (±SD) of mean fluorescence intensity (MFI) of phallodin in the cytoplasm and on the plasma membrane at 10 min (C). About 50 cells from 3 independent experiments using NIS-Elements AR 3.2 software and the MFI of phallodin using flow cytometry (D). Splenic B cells from wild-type (WT) and Rictor KO mice were incubated with AF546-mB-Fab′–anti-Ig tethered to lipid bilayers at 37°C for indicated times. Cells were fixed, permeabilized, and stained. Shown are representative images (E and F) and the average values (± SD) of the MFI of filamentous actin (F-actin) (G), the MFI of the BCR (H), B cell contact area (I), and the MFI of phosphorylated Brutons tyrosine kinase pBtk (J) in the contact zone. Total internal reflection fluorescent microscopy (TIRFm) analysis of the spatial relationship of BCR with phosphotyrosine (pY) and pBtk in the contact zone of splenic B cells incubated with membrane-tethered Fab′–anti-immunoglobulin (Ig). The colocalization coefficients between BCR and pY and pBtk staining were determined using NIS-Elements AR 3.2 software (K). The data were generated using 20–90 cells from 3 independent experiments. Scale bars, 2.5 μm. Mann–Whitney *U* test was used to do the statistics, *p < 0.01. The numerical data (for C, D, G, H, I, J, and K) can be found in S1 Data.

Rictor has been reported in several types of cells to regulate the actin cytoskeleton, although its coordination with actin in lymphocytes still remains elusive [30,31]. Rictor-mTOR complex modulates the phosphorylation of protein kinase C α (PKCα) and the actin cytoskeleton [32]. Our previous studies have shown that actin can offer feedback to BCR signaling [16,33–35]. In order to investigate that the effect of Rictor on BCR signaling is coincident with actin alteration, we examined the Rictor deficiency and actin reorganization in B cells after stimulation with sAgs and membrane-associated antigens (mAgs). Filamentous actin (F-actin) was stained with phallodin, the spatiotemporal position was examined by CFm, and the levels of actin were quantified by NIS-Elements AR 3.2 software and flow cytometry. Compared with the levels of F-actin in WT B cells, the levels of F-actin on the plasma membrane and in the cytoplasm of KO B cells were significantly enhanced at 10 min (Fig 4A–4C). However, the basal levels of F-actin examined by flow cytometry were not altered in KO B cells in the nonstimulated condition (Fig 4D). Interestingly, we found the total levels of F-actin were decreased by 5 min and then increased afterwards until 30 min by flow cytometry (Fig 4D), which indicates the depolymerization of actin in the early phase and polymerization of actin afterwards in WT B cells upon sAg stimulation (Fig 4D). However, we found a dramatic increase of the levels of F-actin by 5 min and a moderate decrease afterwards until 30 min in KO B cells (Fig 4D), and the levels of F-actin in KO B cells were always higher than that of WT B cells (Fig 4D). Because the levels of F-actin were highly condensed on the plasma membrane, we used TIRFm to determine the levels of F-actin in the contact zone between B cells and the antigen-tethered lipid bilayer. In WT B cells, the levels of F-actin on the contact zone were increased over 5 min and decreased at 7 min (Fig 4E and 4G), which is consistent with our previous study [16]. However, in KO B cells, the levels of F-actin were increased over time until 7 min and significantly higher than that of WT B cells (Fig 4E, 4F and 4G). We also determined the recruitment of BCR microclusters in the contact zone by measuring the MFI of the BCR cluster. In WT B cells, the MFI of the BCR cluster was increased over time and it was also increased over time in KO B cells, but it was significantly decreased in KO B cells compared to that of WT B cells (Fig 4E, 4F and 4H). The formation of the BCR microclusters triggers BCR signaling, and we examined the recruitment of activated Btk in the contact zone. The recruitment of pBtk was increased over 5 min and decreased by 7 min in WT B cells, and the recruitment of pBtk in KO B cells had a similar trend but was significantly decreased compared to that of WT B cells (Fig 4J). Our previous research has shown that upon stimulation with mAg, actin polymerizes first to facilitate spreading of B cells and depolymerizes later at the center to promote the formation of the central BCR cluster. During these events, F-actin colocalizes well with BCRs at first and then redistributes to the outer edge of the central BCR cluster [16,35]. As expected, in WT B cells, F-actin colocalized well with BCRs at early time points and redistributed to the outer edge of central BCR cluster (Fig 4E and 4K). The colocalization between BCRs and F-actin increased over 3 min and decreased thereafter (Fig 4E and 4K). In KO B cells, F-actin always colocalized well with BCRs for all the time points analyzed and the colocalization between BCRs and F-actin were increased until 7 min (Fig 4F and 4K). All these results suggest that actin reorganization has been altered in Rictor KO B cells and the absence of Rictor leads to enhanced actin polymerization both in the cytoplasm and on the plasma membrane.

### The enhanced actin polymerization on the B cell plasma membrane restricts BCR movement

**Fig 5:**
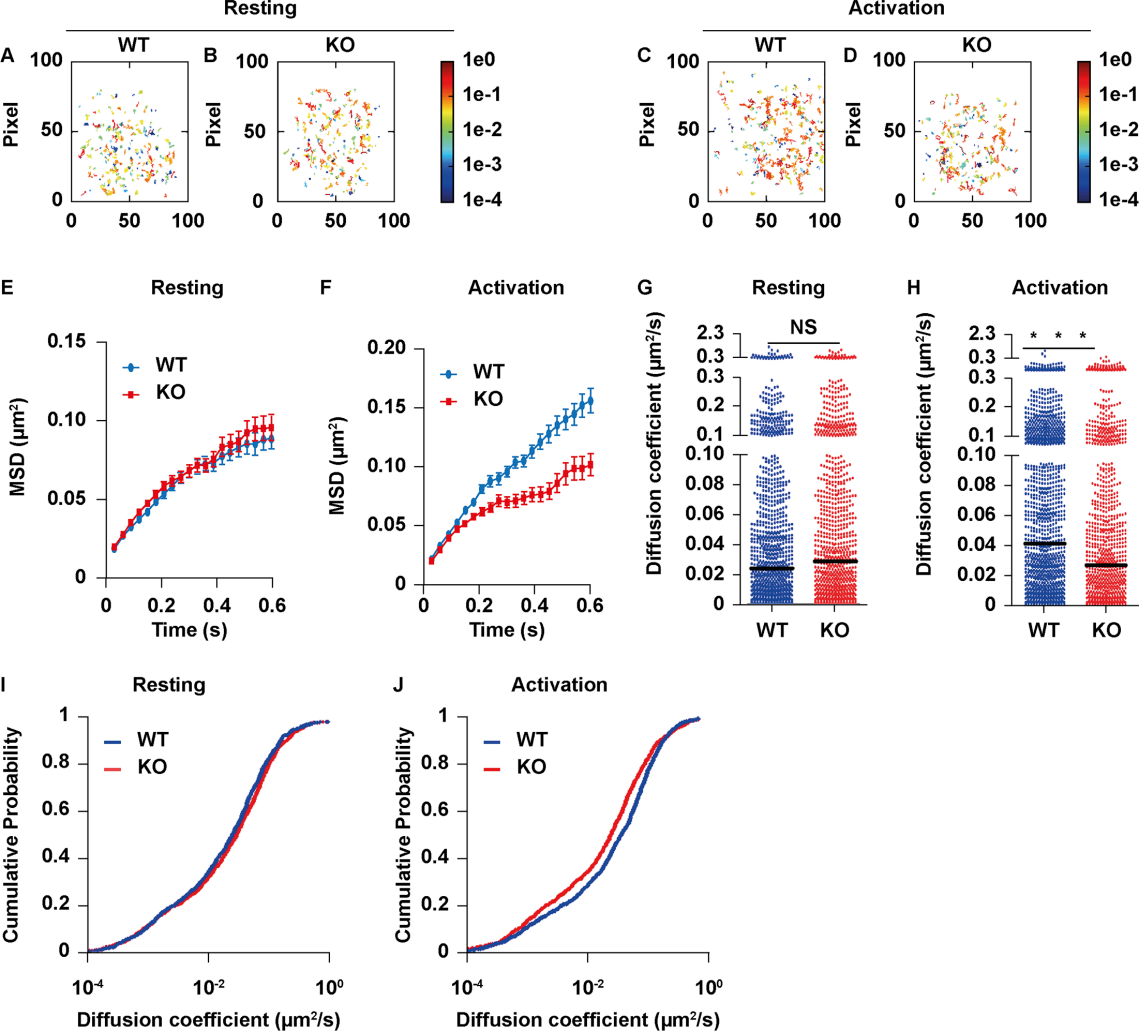
B cell receptor (BCR) mobility is reduced in Rictor knockout (KO) B cells after antigenic stimulation. The primary B cells labeled with low concentration Alexa Fluor 647–Fab anti-immunoglobulin M (IgM) (Fc fragment antibody [Fc] specific) and high concentration Alexa Fluor 647–Fab anti-IgM (Fc specific) was placed on planar lipid bilayers containing anti-major histocompatibility complex (MHC) or anti-IgM, and single BCR molecule total internal reflection fluorescent (TIRF) images were acquired. Individual BCR molecules in TIRF images from 1 typical primary B cell indicate the instant diffusion coefficient (D0) by pseudocolored spots from 5 min to 15 min after stimulation. The display range of the pseudocolor is based on the D0 value of 10–4–0 μm^2^/s, a range that included most of the D0 values of the tracked BCRs. The accumulated trajectory footprints of individual BCR molecules in the entire time course (A–D). All of the D0 values were displayed as mean-square displacement (MSD) plots (E and F), single BCR molecules of the indicated numbers (G and H), and cumulative distribution probability (CDP) plots (I and J) from 3 independent experiments. Mann–Whitney *U* test was used to do the statistics, *p < 0.01. The numerical data (for A, B, C, D, E, F, G, H, I, and J) can be found in S1 Data.

Batista et al. have shown that the actin network restricts the movement of BCRs in the steady state. In the region with higher intensity of actin, the diffusion coefficient was decreased for BCRs [17]. In order to analyze the behavior of a single BCR, we used single-particle tracking and analysis as previously reported [11]. Analyses of the single BCR trajectory footprints suggested that single BCR molecules were more mobile in WT B cells after stimulation than in KO B cells after stimulation with mAg from 5 min to 15 min but no differences in the steady state (Fig 5A–5D). Tracking thousands of single BCR molecules from WT and KO B cells showed that their short-range mean-square displacements (MSDs) did not have differences in the resting state but were significantly decreased in KO B cells after activation (Fig 5E and 5F). The mean diffusion coefficient of the BCRs in KO B cells also decreased significantly during the activation status (Fig 5G and 5H). Moreover, the short-range diffusion coefficients of each individual BCR molecule were calculated and their distribution was analyzed and displayed as a cumulative distribution probability (CDP) plot. The CDP of KO B cells was decreased compared to that of WT B cells upon antigenic mAg stimulation but without changes in the steady state (Fig 5I and 5J). The normal mobility of BCRs in KO B cells for the steady state was consistant with the unchanged basal levels of actin without stimulation (Figs 4D, 5A, 5C, 5E, 5G and 5I). These results imply that the BCRs from the WT and KO B cells almost had the same mobility in the steady state, but the BCRs from KO B cells became less mobile than those of WT B cells after activation. Furthermore, these results suggest that the disrupted actin depolymerization in the early phase and ehanced levels of actin in KO B cells after stimulation with sAg and mAg restrict the movement of BCRs after activation.

### The modest reduction of actin can rescue the defect of BCR signaling in KO B cells

**Fig 6:**
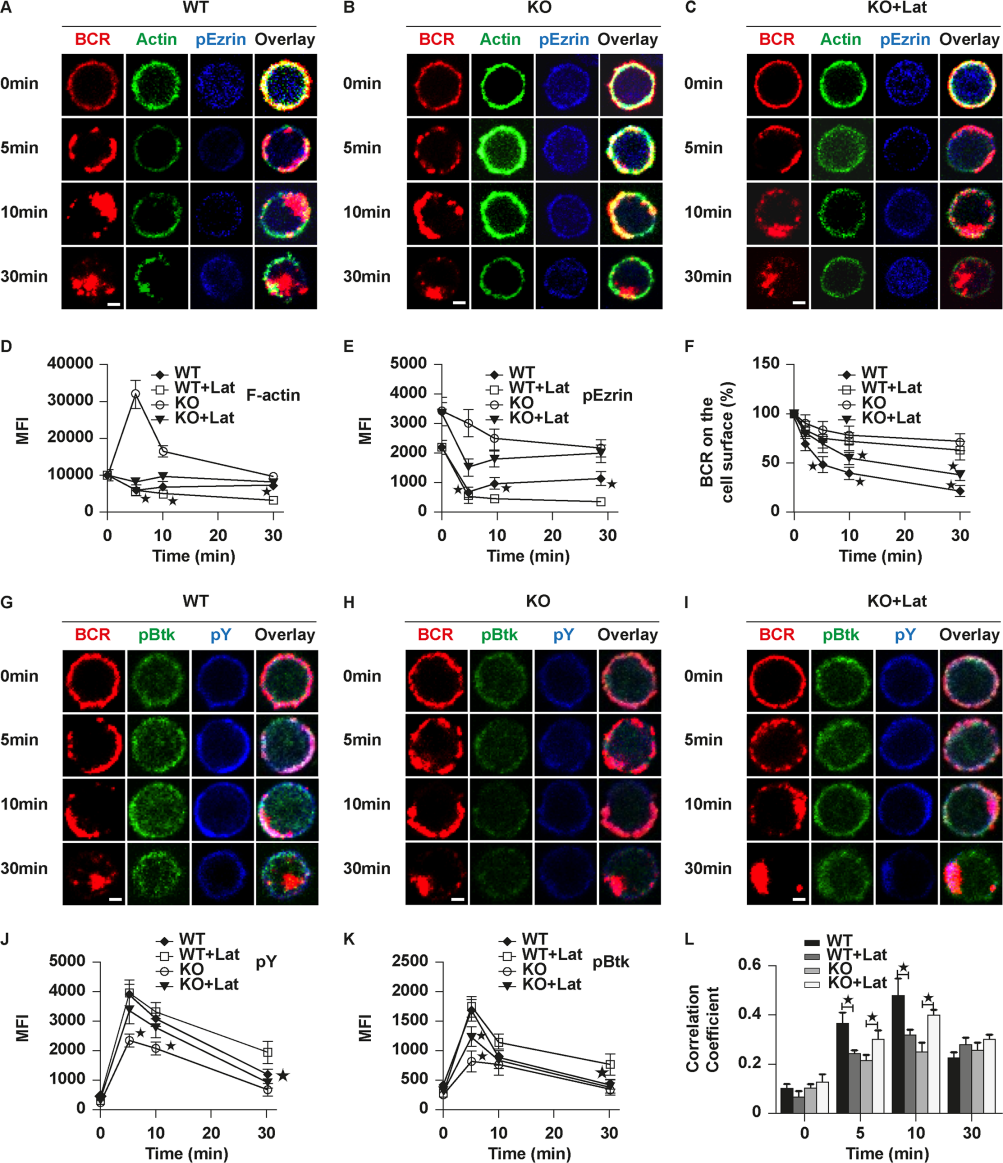
Rictor deficiency deregulates the dephosphorylation of ezrin and actin inhibition can rescue the defects of B cell receptor (BCR) signaling. Splenic B cells were pretreated with or without 0.05 μM Latrunculin B for 30 min and then incubated with Alexa Fluor (AF) 546–monobiotinylated (mB)-Fab′–anti-immunoglobulin (Ig) without (−) or with streptavidin (sAg) at 4°C, washed, and warmed to 37°C for varying lengths of time in the presence of Latrunculin B. (F) Flow cytometry analysis of BCR internalization by quantifying the percentage of biotin-F(ab′)_2_–anti-immunoglobulin (Ig)–labeled BCR remaining on the cell surface after the 37°C chase in the presence of Latrunculin B after treatment. Shown are the average percentages (±SD) from 3 independent experiments. *p < 0.01 compared to control B cells. After fixation and permeabilization, the cells were stained for Alexa Fluor 488 (AF488)-phallodin, phosphorylated Ezrin (pEzrin) (A-C), phosphorylated Brutons tyrosine kinase (pBtk), and phosphotyrosine (pY) (G-I) and analyzed using confocal microscopy (CFm) and flow cytometry (D-F and J-K). The Pearson’s correlation coefficients between BCR and phosphorylated SH2-containing inositol phosphatase (pSHIP) staining in soluble antigen (sAg)-stimulated cells were determined using NIS-Elements AR 3.2 software (L). Shown are representative images at indicated times and the average values (±SD) of about 50 cells from 3 independent experiments. Scale bars, 2.5 μm. One-way ANOVA with the Tukey test was used to do multiple group comparisons, *p < 0.01. The numerical data (for E, F, J, K, and L) can be found in S1 Data.

In order to confirm the effect of actin on BCR signaling in Rictor KO B cells, we used Latrunculin B to reduce the levels of F-actin slightly [15] to see if the defect in BCR signaling and internalization can be rescued. Rictor KO B cells were pretreated with Latrunculin B for 30 min and stimulated with sAg in the presence of Latrunculin B. At 5 min, the levels of F-actin quantified by flow cytometry in KO B cells treated with Latrunculin B were decreased and then gradually increased, which had a similar trend to that of WT B cells, although the levels of F-actin were a little higher (Fig 6A–6D). The levels of F-actin in WT B cells treated with Latrunculin B were decreased compared to that of WT B cells without treatment (Fig 6D). To determine the coordination between actin and ezrin during BCR activation, we stained for activated ezrin by using phosphorylated antibodies. In WT B cells, the levels of phosphorylated Ezrin (pEzrin) decreased for the first 5 min and increased gradually to 30 min (Fig 6A and 6E), which is in line with the previous study [20]. However, in KO B cells, the basal level of pEzrin was significantly higher than that of WT B cells, decreased slowly to 30 min, but was still profoundly higher than that of WT B cells (Fig 6A–6C and 6E). The levels of pEzrin in WT B cells treated with Latrunculin B were decreased compared to those of WT B cells without treatment (Fig 6E). Latrunculin B treatment reduced the activation magnitude of ezrin significantly in KO B cells and induced the same trend as that of WT B cells (Fig 6A–6C and 6E). To further confirm the interplay between actin and ezrin, we used NSC668394 (an ezrin-specific inhibitor) and the inhibitors upstream of the ezrin signaling pathway, such as Y27632 (a ROCK-specific inhibitor) and bisindolylmaleimide I (Bis) (a PKC inhibitor). Not surprsingly, for all 3 inhibitors we found that the actin-polymerization phase starting at 5 min was completely disrupted and replaced with continuous actin depolymerization (S4 Fig). These results collectively suggest that actin and ezrin positively regulate with each other. For BCR internalization, KO B cells treated with Latrunculin B had some BCR caps at 10 min and further flow cytometry analysis found the BCRs remaining on the cell surface decreased significantly compared to that of untreated KO or treated WT B cells but were still higher than that of WT B cells (Fig 6A–6C and 6F). For BCR signaling, the levels of pY and pBtk in KO B cells treated with Latrunculin B increased profoundly compared to those of KO B cells after stimulation (Fig 6G–6K). The levels of pY and pBtk in WT B cells treated with Latrunculin B dropped down more slowly than those of WT B cells without treatment (Fig 6J and 6K). For pY, levels were comparable to those in WT B cells, although the levels of pBtk were still lower than those of WT B cells (Fig 6G–6K). We then looked at the colocalization between BCR, pY, and pBtk. The colocalization between BCR, pY, and pBtk was increased significantly in KO B cells treated with Latrunculin B compared to that of untreated KO B cells but still lower than that of WT B cells (Fig 6G–6I and 6L), and it was decreased in WT B cells treated with Latrunculin B compared to that of untreated WT B cells (Fig 6L). To further confirm that Latrunculin B can rescue the defect of differentiation of FO B cells and BCR signaling of Rictor KO mice *in vivo*, we fed the mice with 0.5 μM Latrunculin B every week for 2 months and then euthanized the mice to analyze the subpopulations and BCR signaling in splenocytes. Latrunculin B treatment largely restored the frequency and number of FO B cells in Rictor KO mice compared to Rictor KO mice treated with vector only but had no effect on the formation of MZ B cells (S5A–S5C Fig). Moreover, the levels of pY or pBtk were also recovered in a large degree in Rictor KO mice treated with Latrunculin B (S5D and S5E Fig). Taken together, these results suggest that enhanced actin polymerization in KO B cells causes the reduction of BCR signaling and differentiation defect of FO B cells.

### The distorted actin reorganization in Rictor KO mice reduces the humoral immune response

**Fig 7:**
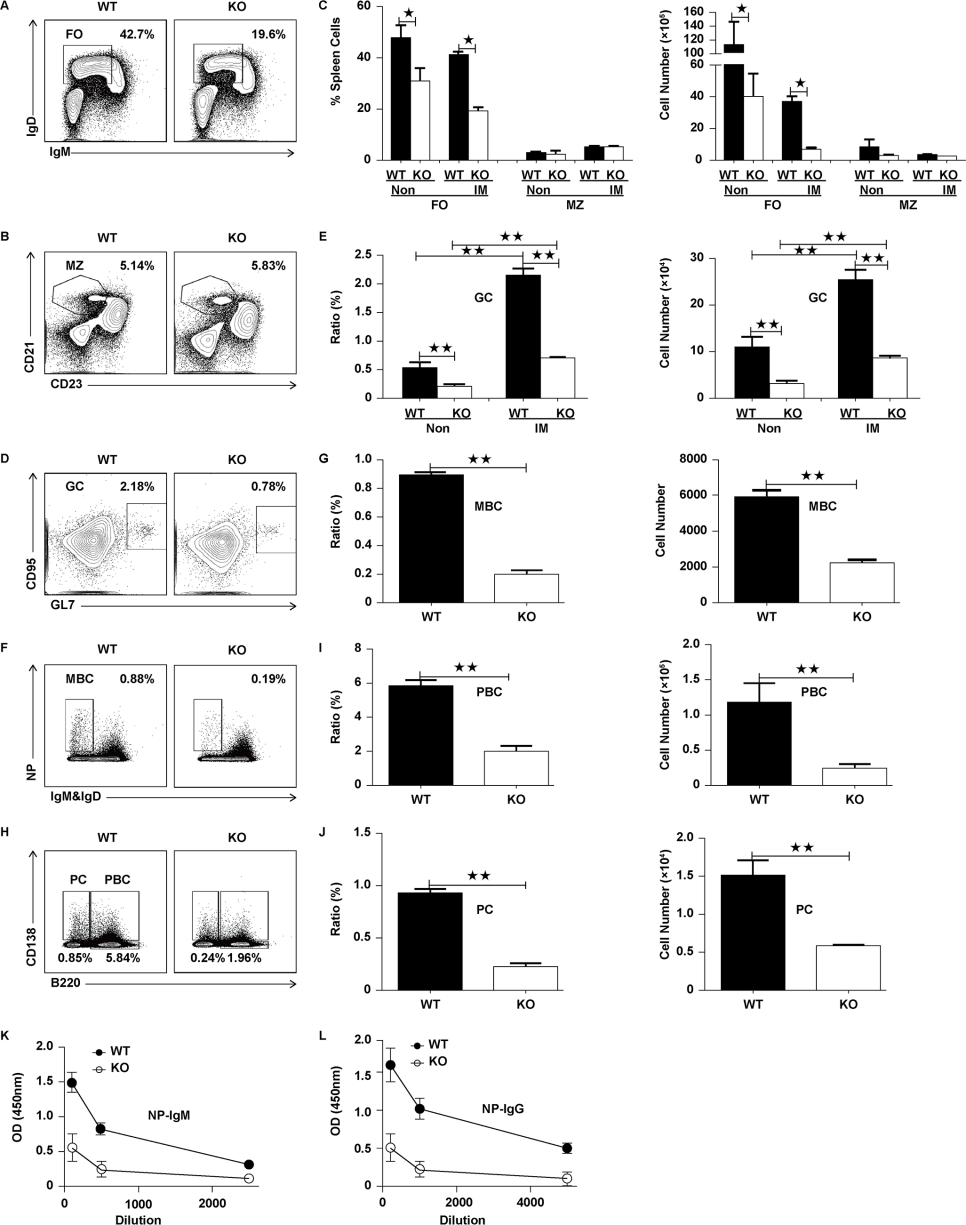
Rictor deficiency reduces the humoral immune response. Flow cytometry analysis of folicular (FO) and marginal zone (MZ) B cells in spleen of wild-type (WT) and Rictor knockout (KO) mice (*n* = 8) immunized with 4-hydroxy-3-nitrophenylacetyl–keyhole limpet hemocyanin (NP-KLH) (A). The quantification of percentage (B) and number (C) of FO and MZ B cells in the spleen of WT and Rictor KO mice. Flow cytometry analysis of germinal center (GC) B cells in the spleen of WT and Rictor KO mice immunized with NP-KLH (D). The quantification of percentage and number of GC B cells in the spleen of WT and Rictor KO mice (E). Flow cytometry analysis of memory B (MBC), plasmablast (PBC), and plasma cell (PC) cells in the spleen of WT and Rictor KO mice immunized with NP-KLH (F and H). The quantification of percentage and number of MBC, PBC, and PC cells in the spleen of WT and Rictor KO mice (G, I, and J). Mean results from ELISA detecting NP-specific immunoglobulin M (IgM) and Immunoglobulin G1 (IgG1) from immunized WT and Rictor KO mice (*n* = 6) (K). T-test was used to do the statistics, **p < 0.001. The numerical data (for C, E, G, I, J, K, and L) can be found in S1 Data.

In order to determine whether the distorted actin reorganization can affect the humoral immune response, we immunized the mice with T-cell dependent antigen–4-hydroxy-3-nitrophenylacetyl–keyhole limpet hemocyanin (NP-KLH). After 14 days, the mice were euthanized and analyzed for several key populations of B cells required for the humoral immune response. We found the percentage and number of FO B cells were profoundly reduced in KO mice after immunization but did not observe any changes for MZ B cells (Fig 7A–7C). Of note, the degree of the reduction of FO B cells in KO mice was greater in immunized mice than that of nonimmunized mice (S3 Fig, Fig 7A and 7C). Furthermore, we analyzed the frequency and number of GC B cells and they were decreased dramatically in KO mice compared to that of WT mice (Fig 7D and 7E). Additionally, we examined the effect of Rictor deficiency on the generation of antigen-specific memory B cells (MBCs); not surprisingly, we found a decrease of the percentage and number of MBCs in immunized KO mice (Fig 7F and 7G). Finally, we examined the plasma cells and plasmablasts in immunized WT and KO mice. We found a significant decrease of plasmablast (PBC) and plasma cell (PC) in immunized Rictor KO mice compared to that of WT mice (Fig 7H and 7J). To further confirm the effect of Rictor deficiency on humoral immune response, we examined the serum levels of NP-specific subclasses from the immunized mice and found the levels of both NP-specific IgM and IgG were decreased in Rictor KO mice (Fig 7K and 7L). Overall, all these results suggest that the distorted actin reorganization contributes to the noncompetent humoral immune response in Rictor KO mice.

## Discussion

This study examined the effect of Rictor deficiency on BCR signaling. We found that the absence of Rictor leads to down-regulation of BCR signaling via decreasing pBtk and increasing pSHIP. Furthermore, the levels of actin are enhanced in both cytoplasm and plasma membrane in Rictor KO B cells stimulated with sAg. Interestingly, the early actin depolymerization phase in WT B cells after stimulation by sAg is replaced with drastically enhanced actin polymerization in Rictor KO B cells. By using the mAg system, we also found an increased level of actin in the contact zone of B cells with the lipid bilayer as well as decreased BCR clustering, B cell spreading, and recruitment of signalosomes in Rictor KO B cells. The increased levels of actin in Rictor KO B cells led to the reduced diffusion coefficient of BCRs in the activation state. Interestingly, we found the phosphorylation of ezrin is increased and the attenuation of phosphorylation is delayed in Rictor KO B cells and that Latrunculin B treatment can rescue the defect of BCR signaling and internalization as well as the FO differentiation. Finally, Rictor deficiency leads to the reduction of FO B cells more severely in immunized mice. Altogether, to our knowledge, this is the first report of how Rictor regulates BCR signaling by altering the actin reorganization via ezrin.

To compare with what has been previously reported, Rictor deficiency causes an impact on the development of bone marrow B cells, although with varying degrees. These differences could be due to the different Cre systems used. Boothby’s group used *Vav*Cre and Yuan’s group used interferon-induced GTP-binding protein Mx1 (*Mx1*)Cre and in both, deletion is in the early stage of B cell development [19,20], and neither of them is B cell specific. In our *cd19-*Cre system, we found a slight impact on the progression of late pre-B cells and recirculating B cells and an increased accumulation of pro-B cells in Rictor KO mice. Boothby’s group also found a slight impact on the pro- and pre-B cells in Rictor KO mice and a profound reduction in MZ B cells that cannot be seen in the *cd19-*Cre KO mice [19]. Yuan’s group found pro-, pre-, and immature B cells are dramatically increased in Rictor KO mice [20]. To resolve the discrepancy between the different Cre systems that have different deletion stages, we are going to use cluster of differentiation 19 (*cd19*)*-*CreER mice to cross with Rictor flox/flox mice to observe any divergence caused by the deletion in different stages besides the deletion in different cells.

Another remaining issue is the detailed link between Rictor and BCR signaling molecules or ezrin. First, it would be interesting to explore the direct interaction between Rictor and BCR signaling molecules such as Btk and SHIP. mTORC2 and the key component, Rictor, specifically, has been shown to phosphorylate Akt and protein kinase B (PKB) on Serine 473 (Ser473). This phosphorylation activates Akt/PKB, whereas dysregulation of Akt/PKB has been correlated with cancer and diabetes [36]. Tyrosine phosphorylation of ezrin regulates the activation of c-Jun N-terminal kinase (JNK) after BCR stimulation [37]. Therefore, Rictor possibly can regulate the phosphorylation of Btk and SHIP. The phosphorylation of ezrin can be regulated by rho-associated coiled-coil-containing protein kinase (ROCK) activation, and additionally mTORC2 has been shown to regulate the actin cytoskeleton through its stimulation of F-actin stress fibers via activation of paxillin, ras homolog family member A (RhoA), ras-related C3 botulinum toxin substrate 1 (Rac1), cell division control protein 42 homolog (Cdc42), and PKCα [32]. Therefore, it would be of interest to explore the possible links between Rictor and the upstream molecules of ezrin, such as rho-associated coiled-coil-containing protein kinase (ROCK), RhoA, or even Wiskott-Aldrich syndrome protein (WASP). Another possibility is the regulation of BCR signaling through transcriptional levels. As a kinase, mTORC2 cannot regulate the genes via transcriptional levels unless it goes through the furthest downstream transcriptional factors such as FoxO1. Therefore, we can examine the mRNA levels of Btk and SHIP as well as other signaling molecules or by microarray to search for other candidate genes, and then to determine whether FoxO1 can regulate these candidate genes using chromatin immunoprecipitation-sequencing (Chip-seq).

In summary, this study has revealed not only a new pathway in BCR signaling but also the detailed molecular mechanism of how Rictor regulates BCR activation. Rictor deficiency leads to dysregulation of dephosphorylation of ezrin, which accounts for the enhanced actin polymerization. The high intensity ezrin-actin areas restrict the movement of BCRs after stimulation, which diminishes the triggering of BCR clustering and consequent BCR signaling. Overall, our study provides a new regulation pathway of Rictor to modulate BCR signaling by the actin-ezrin complex.

## Materials and methods

### Ethics statement

All animal work was reviewed and approved by the Institutional Animal Care and Usage Committee of Children’s Hospital of Chongqing Medical University following institutional and NIH guidelines and regulations.

### Mice and cells

Rictor conditional KO mice on a C57/BL6 background were obtained by crossing *cd*19-Cre mice with *rictor* flox/flox mice from Jackson lab. Splenic B cells were isolated as described previously [38].

### Preparation of monobiotinylated Fab′ Ab and model antigens

Monobiotinylated Fab′ fragment of anti-mouse IgM+G Ab (mB-Fab′–anti-Ig) was made from the F(ab′)_2_ (Jackson ImmunoResearch Laboratories) as described before [39]. The disulfide bond that connects the 2 Fab′ was reduced using 20 mM 2-mercaptoethylamine and then biotinylated by maleimide-activated biotin (Thermo Scientific). Fab′ was purified by using Amicon Ultracentrifugal filters (Millipore) and examined by a biotin quantification kit (Thermo Scientific) and then conjugated with AF546 (Invitrogen). To stimulate B cells with sAg, B cells were incubated with AF546–mB-Fab′–anti-Ig (2 μg/ml) together with mB-Fab′–anti-Ig (8 μg/ml) for 30 min and streptavidin (1 μg/ml) for 10 min at 4°C. Streptavidin was omitted as a negative control. The cells were washed and warmed up to 37°C for different time points. To stimulate B cells with mAg, cells were incubated with AF546–mB-Fab′–anti-Ig and mB-Fab′–anti-Ig tethered to lipid bilayers with streptavidin at 37°C for different time points. As a control, B cells were incubated with AF546–Fab–anti-mouse IgM+G (2 μg/ml) at 4°C and then incubated with transferrin (Tf)-tethered lipid bilayers, on which the density of Tf was equal to that of AF546–mB-Fab′–anti-Ig.

### Preparation of Ag-tethered planar lipid bilayers

The planar lipid bilayer was generated with previous protocol [40,41]. Liposomes were generated by sonicating 1,2-dioleoyl-*sn*-glycero-3-phosphocholine and 1,2-dioleoyl-*sn*-glycero-3-phosphoethanolamine-cap-biotin (Avanti Polar Lipids) in a 100:1 molar ratio in PBS to get 5 mM lipid. Aggregates in liposomes were discarded by ultra centrifugation and filtration. Coverslip chambers (Nalge Nunc International) were incubated with 0.05 mM liposomes for 10 min and then incubated with 1 μg/ml streptavidin (Jackson ImmunoResearch Laboratories) after extensive washes, followed by 2 μg/ml AF546-mB-Fab′–anti-Ig mixed with 8 μg/ml mB-Fab′–anti-Ig Ab.

### TIRFm

Images were obtained using a Nikon A1R confocal and TIRF system on an inverted microscope (Nikon Eclipse Ti-PFS), installed with a 100×, NA 1.49 Apochromat TIRF objective (Nikon Instruments), an iXon EM-CCD camera (Andor), and 3 solid-state lasers with wavelengths 405, 488, and 546 nm.

To image intracellular-signaling molecules, B cells were incubated with an Ag-tethered lipid bilayer at 37°C for different time points. Cells were fixed with 4% paraformaldehyde and permeabilized with 0.05% saponin, followed by phallodin and Btk (pBtk, Y551; BD Biosciences) staining. The B cell contact area and MFI of each staining in the B cell contact zone were determined using IRM images and NIS-Elements AR 3.2 software. Background fluorescence generated by Ag tethered to lipid bilayers in the absence of B cells or secondary Ab controls was subtracted. For each set of data, >20 individual cells from 2 or 3 independent experiments were analyzed. In order to reduce the variability, we consistently dropped cells right above the PBS medium surface using the same volume (10 μl) and cell number (2 x 10^5^) and started timing the early BCR signaling events. We took images from 8 random fields at each time point. We carefully evaluated the morphology and contact area of the B cells landing on the lipid bilayer at different time points. After finishing the analysis of all the individual cells, we pooled all the values together and removed the values that are usually in a very low percentage (<5%) and are far away from the normal range of the majority of the B cells based on the observed morphology and contact area together.

### Confocal and flow cytometry

For confocal analyses, B cells were stimulated with AF546–mB-Fab′–anti-Ig without (−) or with streptavidin (sAg) at 4°C, washed, and warmed to 37°C for different time points. After fixation and permeabilization, the cells were stained for pRictor (T1135, Cell Signaling Technology), pY, pBtk, pSHIP, and pEzrin (T558, Cell Signaling Technology) and analyzed using CFm. For flow cytometric analyses, cell suspensions from BM and spleen were incubated with Fcγ receptor (FcγR) blocking Abs (anti-mouse CD16/CD32; BD Bioscience) on ice and stained at optimal dilutions of conjugated Abs in PBS supplemented with 1% FBS. Anti-mouse Abs and reagents used to stain BM cells included PB-anti-IgM (BioLegend), APC-anti-Ly-51 (BioLegend), PE-anti-CD43 (BioLegend), PerCP-Cy5.5-anti-B220 (BD Bioscience), and PE-Cy7-CD24 (BioLegend) [42,43]. Gating strategy was as follows: A-pre-pro-B cells (BP1^-^CD24^-^), B-pro-B cells (BP1^-^CD24^+^), C-early pre-B cells (BP1^+^CD24^+^), D-late pre-B cells (B220^+^IgM^-^), E-immature B cells (B220^Int^IgM^+^), and F-recirculating B cells (B220^high^IgM^+^) included BV510-anti-IgD (Southern Biotech), FITC-anti-B220 (BioLegend), and PB-anti-IgM (BD Biosciences) [44]. Gating strategy was as follows: FO B cells (B220^high^ IgM^low^ IgD^high^), MZ B cells (B220^high^ CD21^high^CD23^low^). Anti-mouse Abs and reagents used to stain splenic MZ B cells included APC-anti-CD21 (BioLegend), FITC-anti-B220, and PE-anti-CD23 (BD Biosciences) [44]. Anti-mouse Abs and reagents to stain splenic GC B cells included FITC-anti-CD95 (BD Biosciences), APC-anti-GL7, and PerCP-Cy5.5-anti-B220 (BD Biosciences) [45]. Anti-mouse Abs and reagents to stain splenic MBC, PBC, and PC B cells included FITC-anti-CD95 (BD Biosciences), Pac-Blue-anti-GL7 (Biolegend), BV510-anti-B220 (Biolegend), NP-PE (Biosearch Technologies), APC-anti-CD138 (Biolegend), PE-Cy7-anti-IgD (Biolegend), and PE-Cy7-anti-IgM (Biolegend). Anti-mouse Abs and reagents used to treat B cells for BCR signaling include: FITC-anti-B220. B cells were stimulated with F(ab′) -anti-Ig plus streptavidin (Jackson ImmunoResearch) at 37°C. The cells were fixed, permeabilized, and stained with pY (Millipore), phosphorylated Btk (pBtk, Y551; BD Biosciences), phosphorylated SHIP (pSHIP, Y1020; Cell Signaling Technology), phallodin, phosphorylated ezrin (pEzrin, T558; Cell Signaling Technology), phosphorylated Erk (pErk, T202/Y204; BD Biosciences), phosphorylated Akt (pAkt, S473;BD Biosciences). Stained cells were analyzed by a BD FACS Canto and analyzed using FlowJo software (Tree Star).

### Immunoblotting

Splenic B cells were incubated with mB-Fab′–anti-Ig without (−) or with streptavidin (sAg) at 4°C, washed, and warmed to 37°C for indicated times and lysed. Cell lysates were analyzed with SDS-PAGE and western blot and probed for pAkt (Ser473; Cell Signaling Technology), pERK1/2 (T202/Y204; Cell Signaling Technology), pBtk (pBtk, Y551; BD Biosciences), and pY (Millipore). Anti-mouse β-actin Ab (Sigma-Aldrich) was used for loading controls.

### Quantitative RT-PCR

For comparison of *rictor* gene expression in WT and Rictor KO B cells, RNA was isolated with RNAPURE kit (RP1202; BioTeke) and reverse transcribed with a PrimeScript RT reagent Kit (RR037A; Takara). The transcribed cDNA was used to analyze the expression of different genes with SsoAdvanced SYBR Green supermix (Bio-Rad) on a CFX96 Touch Real-Time System (Bio-Rad). *rictor* 5’primer:tgcgatattggccatagtga and 3’primer: acctcgttgctctgctgaat.

### Immunization and ELISA

WT and Rictor KO mice were bred and maintained in a specific-pathogen–free animal facility. All mice were male and aged 6–8 weeks. For NP-KLH immunization, 400 μg NP-KLH (Biosearch Technologies) in 400 μl Ribi Adjuvant (MPL+TDM Adjuvant System; Sigma) was injected in the flank subcutaneously at day (d) 1. At d 14 after immunization, the spleen was harvested and immune cells were isolated by sucrose density centrifugation using Lymphocyte Separation Media (LSM; MPbio). For detection of serum levels of NP-specific subclasses, mice were immunized and boosted with the same 2 week later. Serum collected 5 d after the boost (19 d after primary immunization) was analyzed by ELISA using NP-bovine serum albumin–coated plates and Ig isotype specific secondary Ab (Southern Biotech).

### Inhibitors treatment *in vitro* and *in vivo*

B cells were pretreated with 0.05 μM Latrunculin B, 1 μM Bis, 10 μM NSC668394, or 10 μM Y27632 (Calbiochem, Gibbstown, NJ) for 30 min at 37°C before stimulation with Ag in the presence of inhibitors. Mice were fed with 0.5 μM Latrunculin B by IP injection every week for 2 months [46].

### BCR internalization

Splenic B cells were stimulated with biotinylated F(ab′)_2_-goat anti-mouse IgG+M (10 μg/ml; Jackson ImmunoResearch) at 4°C and pulsed at 37°C. Biotin-F(ab′)_2_–anti-IgG+M remaining on the cell surface after the stimulation was stained with PE-streptavidin and examined by flow cytometry. The data were shown as percentages of the cell-surface–associated biotin-F(ab′)_2_–anti-IgG+M at time 0.

### Single-molecular tracking and analysis

Single BCR–molecule imaging was performed according to previous protocol [47]. In detail, prelabeled WT and Rictor KO B cells were imaged by TIRFm with a 640-nm laser in the epifluorescence mode at an output power of 10 mW at the objective lens. A region of 100 ×100 pixels of the area of the electron-multiplying CCD chip was used to obtain an exposure time of 30 ms/frame, the time resolution of which was enough to track the single-molecule BCRs as published [11,47]. Single-molecule tracking of BCR molecules was analyzed as described before [11,47]. Short-range diffusion coefficients and MSD for each BCR molecule trajectory were determined and plotted as CDPs from positional coordinates.

### Calcium flux

The level of calcium flux was examined by flow cytometry using the calcium-sensitive dyes Fluo4 AM and Fura Red (Life) according to the established protocols. The relative levels of intracellular calcium flux were measured by a ratio of Fluo4 to Fura Red emission using FlowJo software (Tree Star, Inc., Ashland, OR) [34].

### Statistical analysis

Statistical significance was assessed using t-test or the Mann–Whitney *U* test. When multiple groups were compared, 1-way ANOVA with the Tukey test was performed (GraphPad Software, San Diego, CA). The *p* values were determined in comparison with WT or control B cells. * *p* < 0.01, ** *p* < 0.001.

## Supporting information

S1 Fig CD19cre has specific deletion in B cells. Real-time PCR analysis of rictor mRNA expression in fresh isolated B cells, CD4^+^ and CD8^+^ T cells (A). Western blot analysis of Rictor expression in fresh isolated B cells (B). Shown are the results from three independent experiments. T-test was used to do the statistics,*p < 0.01. The numerical data (for A) can be found in S1 Data.
(TIF)

S2 Fig Rictor KO deficiency has moderete effect on the development of bone marrow B cells. Cells from BM of WT and Rictor KO mice (n = 6) were labeled with Abs specific for surface markers of prepro- (A), pro- (B), early pre- (C), late pre- (D), immature (E), and recirculating mature B cells (F) and CD127 in the BM, and analyzed using flow cytometry. Shown are representative dot plots (A and B), the average percentages (+SD) and numbers of cells extracted from BM (C and D), the average MFI of CD127 in different B-cell subsets (E). T-test was used to do the statistics,*p < 0.01.The numerical data(for C, D and E) can be found in S1 Data.
(TIF)

S3 Fig Rictor KO deficiency has impact on the differentiation of FO and GC B cells. B cells from non-immunized WT and Rictor KO mice (n = 8) were stained with labeled Abs specific for surface markers of FO, MZ and GC B cells. Then samples were analyzed by flow cytometry. Shown are representative dot plots (A-C), the average percentages (+SD) and numbers of cells extracted from spleen (D-G) of three independent experiments and the MFI of IgD and IgM expression in B220^+^ B cells (H and I). T-test was used to do the statistics,*p < 0.01.The numerical data(for D, E, F, G, H and I) can be found in S1 Data.
(TIF)

S4 Fig Ezrin phosphorylation inhibition ablolishes the actin accumulation in the late stage. Splenic B cells were pretreated with or without Y27632, Bis or NSC668394 for 30 min and then incubated with mB-Fab′–anti-Ig without (−) or with streptavidin (sAg) at 4°C, washed, and warmed to 37°C for varying lengths of time in the presence of inhibitors. After fixation and permeabilization, the cells were stained for AF488-phallodin analyzed using flow cytometry. One-way ANOVA with the Tukey test was used to do multiple group comparisons, *p < 0.01.The numerical data can be found in S1 Data.
(TIF)

S5 Fig Latruculin.B treatment restores the differentation of FO B cells and BCR signaling in Rictor KO mice *in vivo*. Splenic B cells from WT mice treated with vector (WT), KO mice treated with vector (KO) and KO mice (n = 9) treated with Latrunculin.B (KO+Lat) were analyzed by flow cytometry with specific markers for FO and MZ B cells. Shown are the representative dot plots (A) and the average percentages (+SD) and numbers of cells extracted from spleen (B-C) of three independent experiments. Splenic B cells from WT mice treated with vector (WT), KO mice treated with vector (KO) and KO mice treated with Latrunculin.B (KO+Lat) were stimulated with sAgs, stained with antibodies specific for pY or pBtk and analyzed with flow cytometry (D-E). Shown are the results from three independent experiments, One-way ANOVA with the Tukey test was used to do multiple group comparisons,*p < 0.01.The numerical data(for B, C, D and E) can be found in S1 Data.
(TIF)

S1 Data Data for figures and supporting. All data used to create the figures in the manuscript.
(XLS)

## Abbreviations

AF546–mB-Fab′–anti-Ig: Alexa Fluor 546–monobiotinylated-Fab′–anti-immunoglobulin
BCR: B cell receptor
Bis: bisindolylmaleimide I
CD3: cluster of differentiation 3
CD19: cluster of differentiation 19
Cdc42: cell division control protein 42 homolog
CDP: cumulative distribution probability
CFm: confocal microscopy
Chip-seq: chromatin immunoprecipitation-sequencing
D0: instant diffusion coefficient
ERM: ezrin-radixin-moesin
F-actin: filamentous actin
FcγR: Fc gamma receptor
FO: folicular
FoxO1: forkhead box O1
GC: germinal center
Igα: immunoglobulin α chain
Igβ: immunoglobulin β chain
IgD: immunoglobulin D
IgM: immunoglobulin M
IL-7: interleukin 7
ITAM: immunoreceptor tyrosine-based activation motif
JNK: c-Jun N-terminal kinase
KLH: keyhole limpet hemocyanin
KO: knockout
Lyn: LYN proto-oncogene, Src family tyrosine kinase
mAg: membrane-associated antigen
MBC: memory B cell
MFI: mean fluorescence intensity
MHC: major histocompatibility complex
MSD: mean-square displacement
mTORC: mammalian target of rapamycin complex
mTORC2: the mammalian target of rapamycin complex 2
Mx1: interferon-induced GTP-binding protein Mx1
MZ: marginal zone
NF-κB: nuclear factor κB
NP: 4-hydroxy-3-nitrophenylacetyl
pBtk: phosphorylated Brutons tyrosine kinase
PBC: plasmablast
PC: plasma cell
pErk: phosphorylated extracellular regulated protein kinases
pEzrin: phosphorylated Ezrin
PKB: protein kinase B
PKC: protein kinase C
PKCα: protein kinase C α
PI3K: phosphotidylinositol 3 kinase
PLCγ: phospholipase C gamma 2
pRictor: phosphorylated Rictor
pSHIP: phosphorylated SH2-containing inositol phosphatase
pY1: phosphotyrosine
Rac1: ras-related C3 botulinum toxin substrate 1
Rag-1: recombination activating 1
RhoA: ras homolog family member A
ROCK: rho-associated coiled-coil-containing protein kinase
RT-PCR: real time PCR
sAg: soluble antigen
Ser473: Serine 473
siRNA: small interfering RNA
SHIP: SH2-containing inositol phosphatase
SLE: systemic lupus erythematosus
Syk: spleen tyrosine kinase
TCR: T cell receptor
TIRFm: total internal reflection fluorescent microscopy
Vav: vav guanine nucleotide exchange factor
WASP: Wiskott-Aldrich syndrome protein
WT: wild-type
