# Supplementary figures and images for: Rictor positively regulates B cell receptor signaling by modulating actin reorganization via ezrin
Source: PLoS Biol. 2017 Aug 18;15(8):e2001750. doi: 10.1371/journal.pbio.2001750 (PMC5562439; doi:10.1371/journal.pbio.2001750)

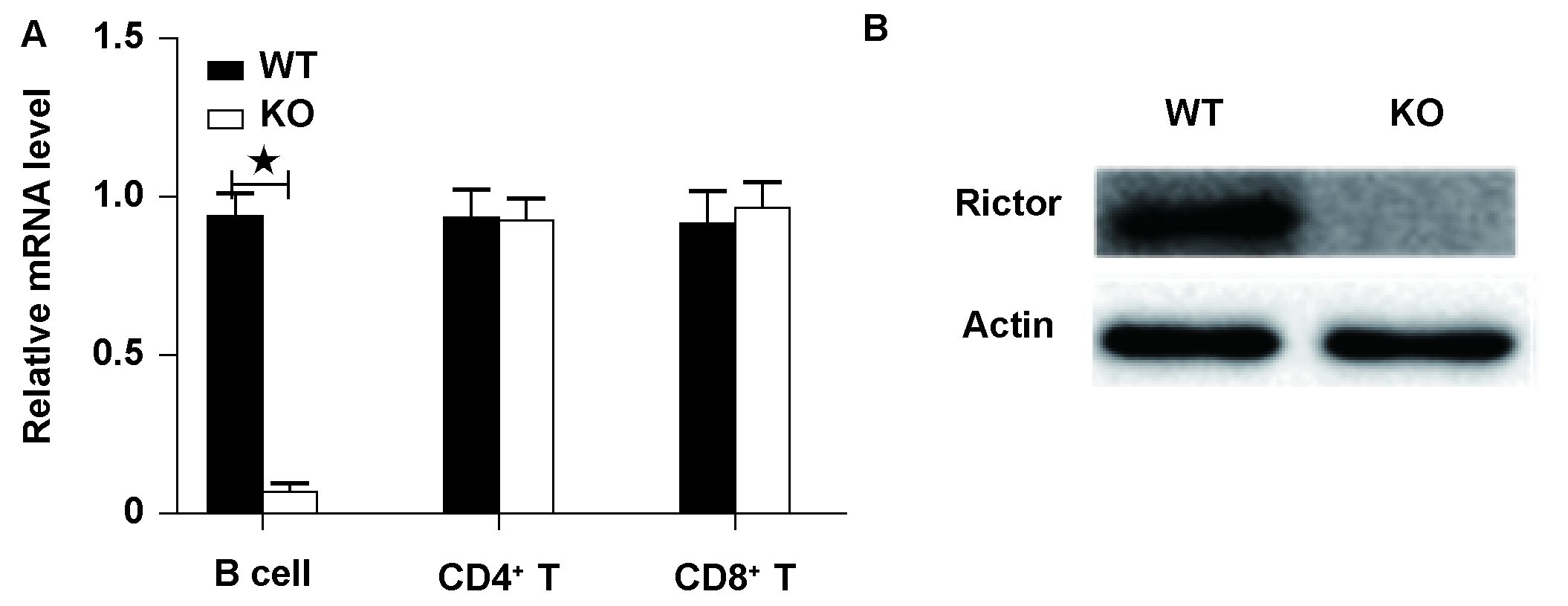

Supplement: S1 Fig — Real-time PCR analysis of rictor mRNA expression in fresh isolated B cells, CD4+ and CD8+ T cells (A). Western blot analysis of Rictor expression in fresh isolated B cells (B). Shown are the results from three independent experiments. T-test was used to do the statistics,*p < 0.01. The numerical data (for A) can be found in S1 Data. (TIF) [file pbio.2001750.s001.tif]

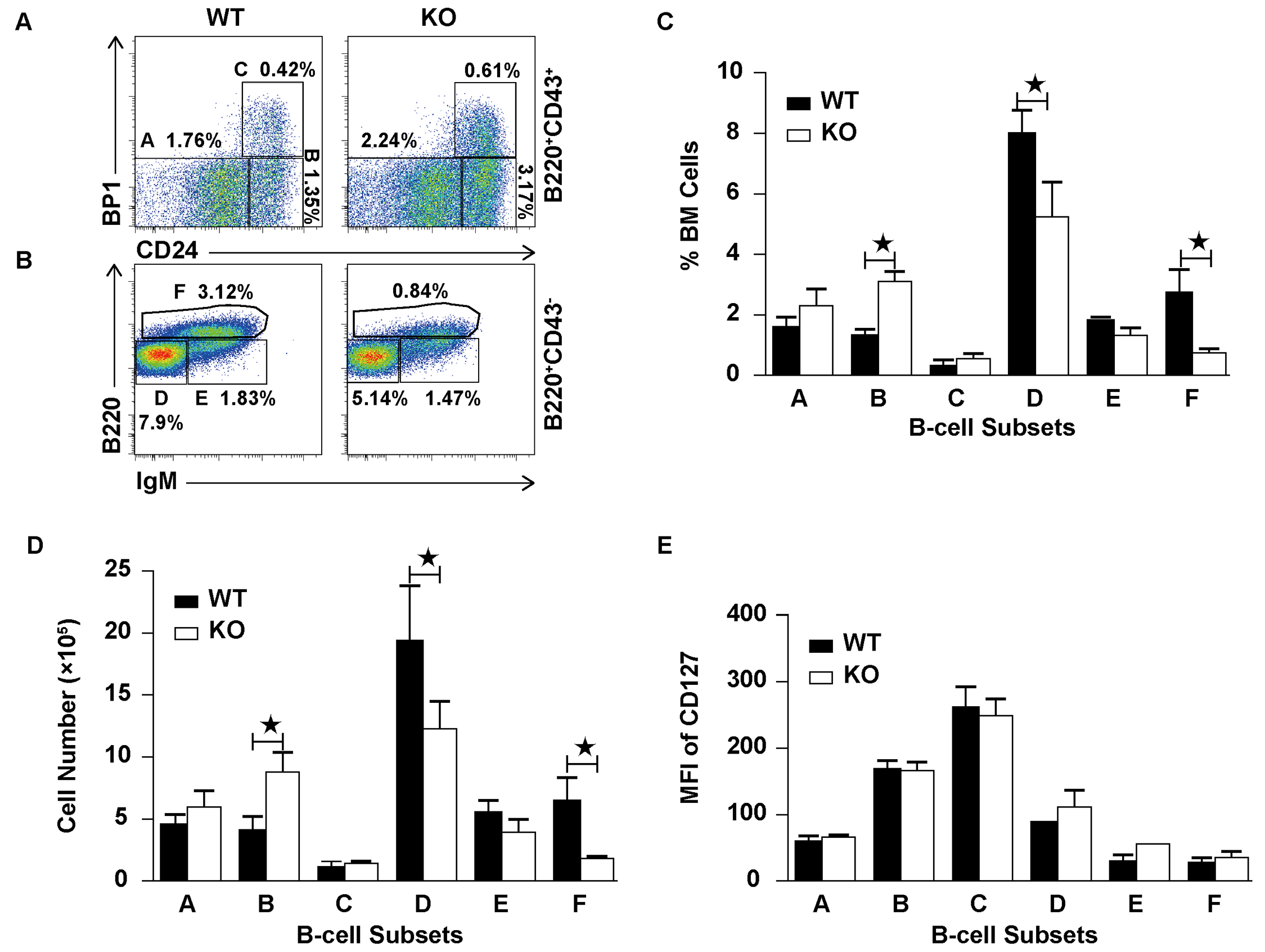

Supplement: S2 Fig — Cells from BM of WT and Rictor KO mice (n = 6) were labeled with Abs specific for surface markers of prepro- (A), pro- (B), early pre- (C), late pre- (D), immature (E), and recirculating mature B cells (F) and CD127 in the BM, and analyzed using flow cytometry. Shown are representative dot plots (A and B), the average percentages (+SD) and numbers of cells extracted from BM (C and D), the average MFI of CD127 in different B-cell subsets (E). T-test was used to do the statistics,*p < 0.01.The numerical data(for C, D and E) can be found in S1 Data. (TIF) [file pbio.2001750.s002.tif]

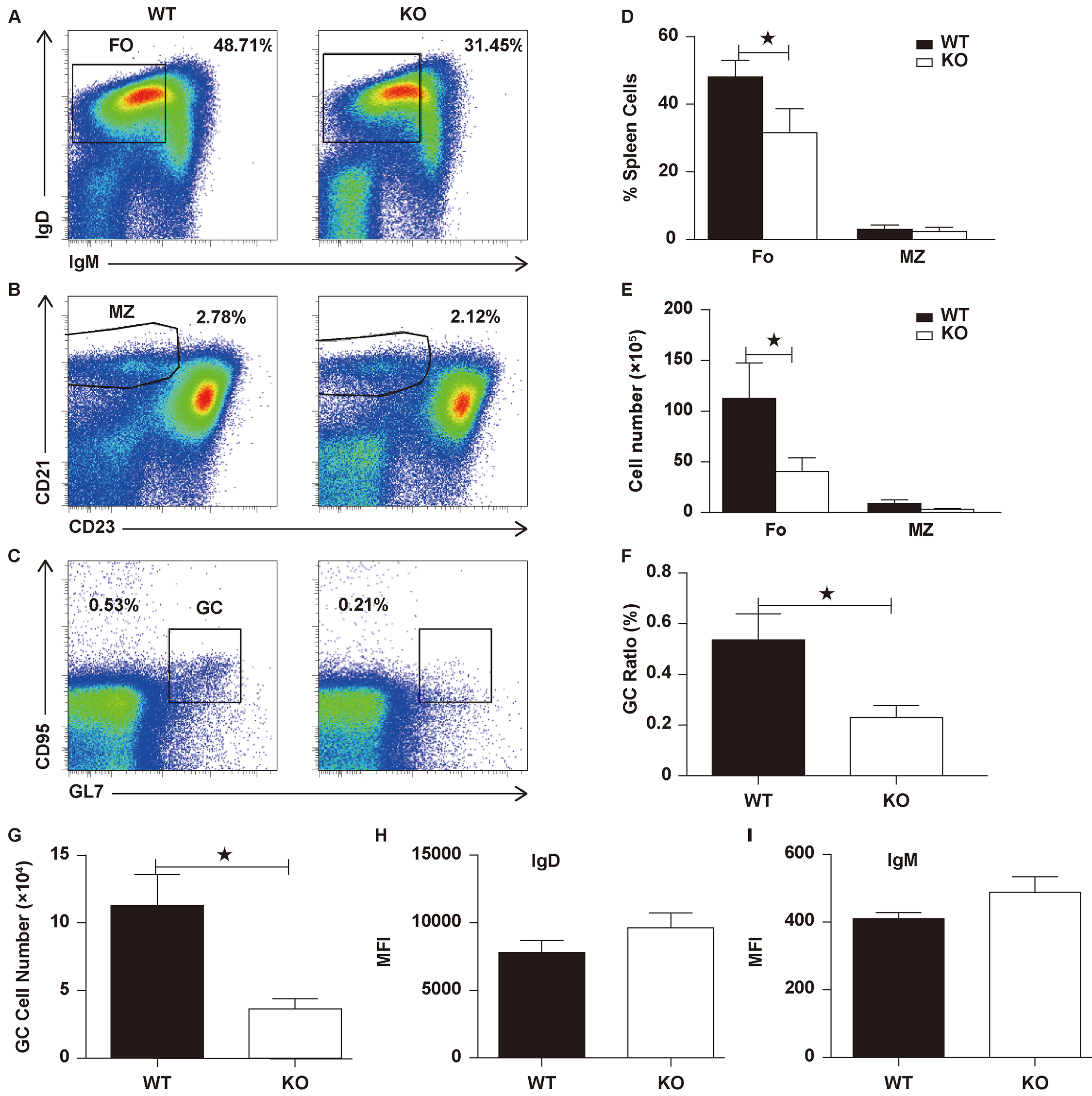

Supplement: S3 Fig — B cells from non-immunized WT and Rictor KO mice (n = 8) were stained with labeled Abs specific for surface markers of FO, MZ and GC B cells. Then samples were analyzed by flow cytometry. Shown are representative dot plots (A-C), the average percentages (+SD) and numbers of cells extracted from spleen (D-G) of three independent experiments and the MFI of IgD and IgM expression in B220+ B cells (H and I). T-test was used to do the statistics,*p < 0.01.The numerical data(for D, E, F, G, H and I) can be found in S1 Data. (TIF) [file pbio.2001750.s003.tif]

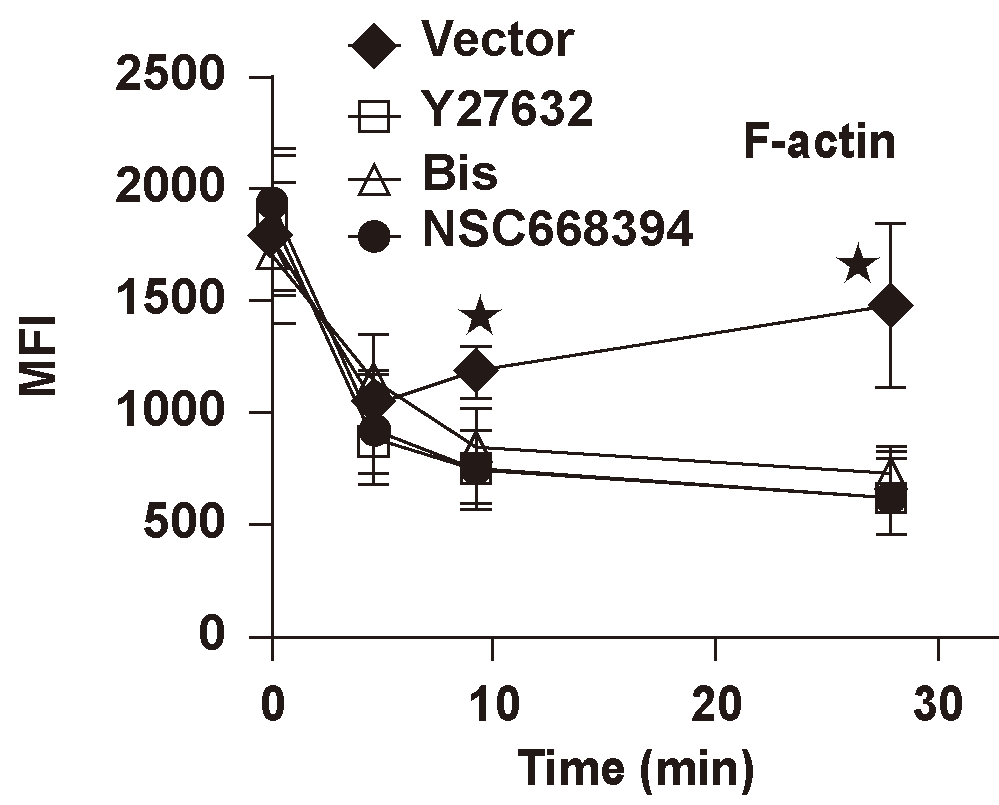

Supplement: S4 Fig — Splenic B cells were pretreated with or without Y27632, Bis or NSC668394 for 30 min and then incubated with mB-Fab′–anti-Ig without (−) or with streptavidin (sAg) at 4°C, washed, and warmed to 37°C for varying lengths of time in the presence of inhibitors. After fixation and permeabilization, the cells were stained for AF488-phallodin analyzed using flow cytometry. One-way ANOVA with the Tukey test was used to do multiple group comparisons, *p < 0.01.The numerical data can be found in S1 Data. (TIF) [file pbio.2001750.s004.tif]

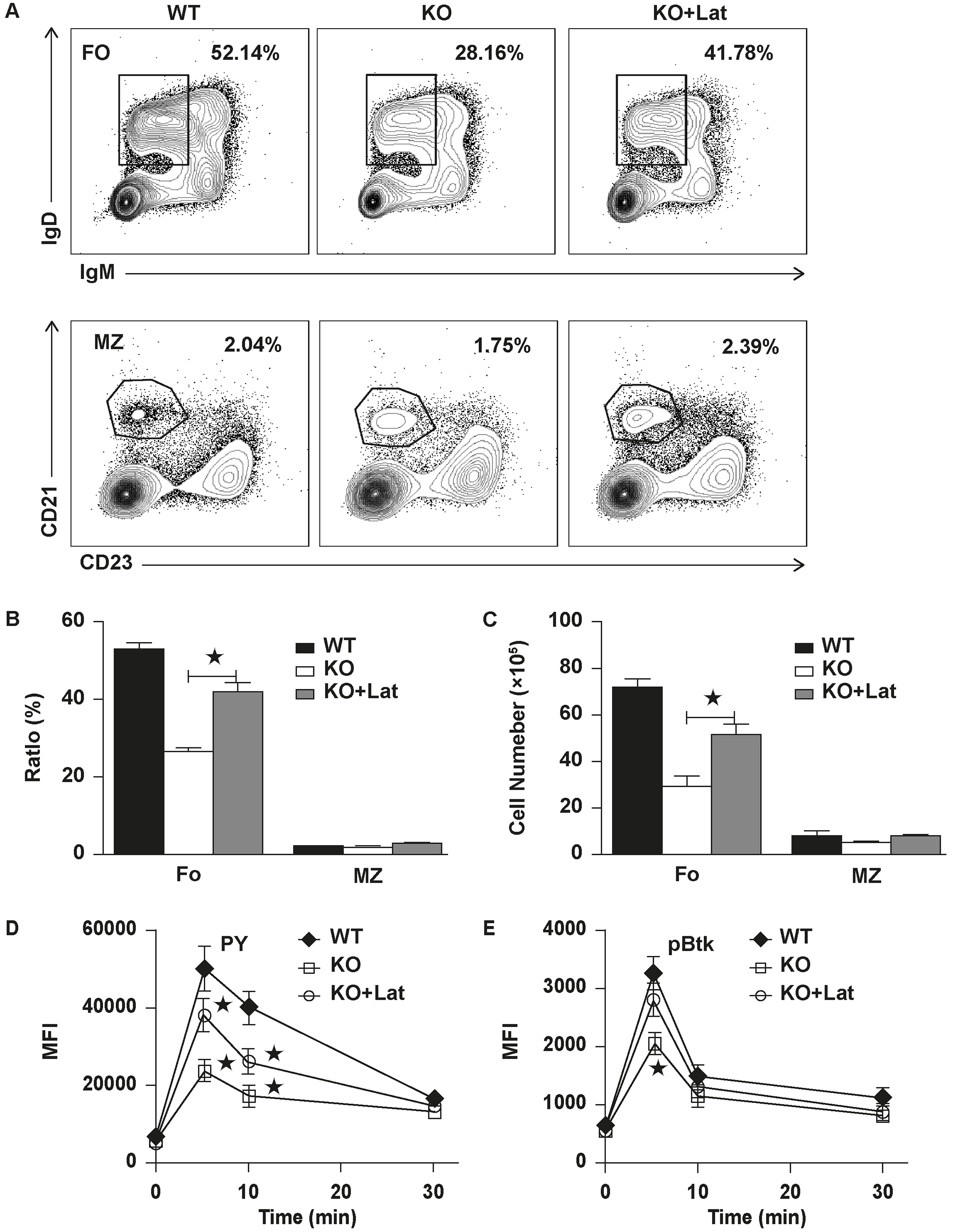

Supplement: S5 Fig — Splenic B cells from WT mice treated with vector (WT), KO mice treated with vector (KO) and KO mice (n = 9) treated with Latrunculin.B (KO+Lat) were analyzed by flow cytometry with specific markers for FO and MZ B cells. Shown are the representative dot plots (A) and the average percentages (+SD) and numbers of cells extracted from spleen (B-C) of three independent experiments. Splenic B cells from WT mice treated with vector (WT), KO mice treated with vector (KO) and KO mice treated with Latrunculin.B (KO+Lat) were stimulated with sAgs, stained with antibodies specific for pY or pBtk and analyzed with flow cytometry (D-E). Shown are the results from three independent experiments, One-way ANOVA with the Tukey test was used to do multiple group comparisons,*p < 0.01.The numerical data(for B, C, D and E) can be found in S1 Data. (TIF) [file pbio.2001750.s005.tif]
